# Test-Retest Reliability and Sensitivity of Kinematic and Kinetic Metrics Measured from Horizontal Deceleration Ability Tests with Different Sprinting Distances

**DOI:** 10.5114/jhk/189659

**Published:** 2024-12-06

**Authors:** Zhili Chen, Mengde Lyu, Mingyue Yin, Shengji Deng, Chris Bishop, Damian Harper, Boyi Dai, Yongming Li

**Affiliations:** 1 School of Athletic Performance, Shanghai University of Sport, Shanghai, China.; 2 Faculty of Science and Technology, London Sport Institute, Middlesex University, London, United Kingdom.; 3 Institute of Coaching and Performance, School of Health, Social Work and Sport, University of Central Lancashire, Preston, United Kingdom.; 4 Division of Kinesiology and Health, University of Wyoming, Laramie, United States.; 5 China Institute of Sport Science, Beijing, China.

**Keywords:** repeatability, braking, athlete, field testing, performance

## Abstract

This study aimed to (1) assess the test-retest reliability and sensitivity of kinematic and kinetic metrics from 5-m, 10-m, and 20-m horizontal deceleration ability (HDA) tests; (2) explore the relationships of those metrics from HDA5m, HDA10m and HDA20m tests. Eighteen college athletes completed one familiarization session and two test sessions separated by 48 hours. Test sessions consisted of three 5-m, 10-m, and two 20-m maximal sprints, along with HDA tests conducted at the same sprinting distances. The deceleration-related metrics measured from HDA5m, HDA10m and HDA20m tests showed good-to-excellent overall reliability (ICC > 0.75, CV < 5.81%) and were efficiently sensitive in detecting moderate changes in deceleration performance (SEM < SWC0.5), except for the DTS and TTS of the HDA20m test (ICC: 0.44–0.57, CV: 5.15–6.37%, SEM > SWC0.5). DTS and TTS of the HDA5m test showed non-significant and small to moderate relationships with the HDA10m and HDA20m tests, while all kinetic metrics displayed significant and large to very large correlations among three tests. This suggests that short-distance HDA tests are reliable and sensitive for assessing deceleration performance, and further research is needed to explore the biomechanical and physiological factors influencing this unique ability.

## Introduction

Deceleration can be mechanically defined as “the decreasing velocity with respect to time” (Winter et al., 2016). For athletes participating in team sports (such as basketball, soccer, rugby), deceleration is a crucial quality for performing rapid changes of direction that are required to successfully evade or pursue an opponent (Delaney et al., 2017; Forster et al., 2023; Koyama et al., 2022) in order to gain a competitive advantage (Ciocca et al., 2021; Harper et al., 2022a; Hewit et al., 2011). Harper et al. (2019) reported that high-intensity decelerations (< −2.5 m/s^2^) occur more frequently than equivalent intensity accelerations (> 2.5 m/s^2^) during the majority of team sports competitions. Numerous research studies have focused on improving athletes' acceleration ability (Bielec et al., 2013; Morris et al., 2022; Nicholson et al., 2021; Wild et al., 2018), culminating in a comprehensive understanding of how to measure and evaluate this physical ability (Haugen and Buchheit, 2016) and the associated training methods (Hicks et al., 2020; Tønnessen et al., 2011). By comparison, there is a distinct lack of literature related to athlete’s deceleration ability, resulting in a comparably reduced level of understanding of this physical quality (Harper et al., 2019, 2022a). Given that fast horizontal decelerations impose higher mechanical loads compared to accelerations (Harper and Kiely, 2018; Harper et al., 2022a), developing our ability to better understand this component of performance is crucial for developing training strategies focused on athletic enhancement and injury prevention.

Thus far, horizontal deceleration ability (HDA) has been qualified using an acceleration-to-deceleration test whereby the athlete sprints across a pre-set distance before performing a maximal deceleration (Ashton and Jones, 2019; Graham-Smith et al., 2018; Harper et al., 2023). Radar or laser devices are used to obtain instantaneous measures of time, velocity, and distance throughout the acceleration and deceleration phases to then enable calculation of deceleration kinetic (i.e., braking force, power, and impulse) and/or kinematic (i.e., average deceleration, distance-to-stop, time-to-stop) variables. Nevertheless, few studies have investigated the repeatability of this approach to evaluate the kinetic and kinematic variables indicative of an athlete’s HDA (Ashton and Jones, 2019; Harper et al., 2023). Ashton and Jones (2019) measured deceleration distances using a laser device at 75% to 0% of individual's maximal 15-m sprint velocity in 20 amateur rugby union players. Based on the within and between session reliability data, the authors recommended using the total deceleration distance-to-stop to quantify deceleration ability. However, the coefficient of variation (CV%) for this variable was 10.52%, suggesting that further work is needed to create a protocol that is more sensitive to monitoring changes in deceleration performance. Afterward, Harper et al. (2023) specifically developed an acceleration-deceleration ability test protocol. In this test, the athlete is required to sprint 20-m first and then perform maximal horizontal deceleration. The time point immediately following the maximum velocity achieved during the 20-m sprint was defined as the start of the deceleration phase. Using this protocol and a radar device, the authors reported good intra-day and inter-day reliability (ICC > 0.75, CV < 10%) for a range of deceleration performance related metrics (Harper et al., 2023).

However, it is worth noting that the deceleration phase in the aforementioned studies was performed following a sprint of between 15 to 20 m, yet no research has determined the reliability and sensitivity of HDA tests performed with shorter sprint distances (i.e., 5 m and 10 m). This is a potential limitation due to the fact that athletes in many team-based court sports primarily perform decelerations primarily from shorter sprinting distances and speeds. For example, sprints of basketball players during competition averaged 0.5 to 2.0 s in duration, covering distances of approximately 3 to 9 m with the majority of sprints less than 20 m in length (Wen et al., 2018). In futsal, the mean sprint duration and distances have been reported to be from 1.6 to 1.9 s and 7.8 to 13.0 m, respectively (Taylor et al., 2017). Furthermore, even in soccer, the frequency of long-distance sprints (> 20 m) varies by position, with forwards and external midfielders performing more sprints longer than 20 m compared to central midfielders and central defenders (6.9 to 7.4 vs. 3.4 to 4.1) (Andrzejewski et al., 2013). It follows that if the HDA test involves too long sprint distances, it might lack ecological validity for athletes who perform more frequently decelerations after shorter sprint distances. Furthermore, deceleration performance evaluated from higher sprinting speeds may not be reflective of deceleration performance performed from lower sprinting speeds (Philipp et al., 2023a). With this in mind, there is also a need to investigate relationships between deceleration performance variables performed with different sprinting distances and speeds.

Accordingly, this study aimed to (1) assess the test-retest reliability and sensitivity of deceleration-related kinetic and kinematic metrics from HDA tests with shorter to longer sprinting distances (i.e., 5 m, 10 m and 20 m), and (2) explore relationships among the deceleration-related metrics measured from HDA_5m_, HDA_10m_ and HDA_20m_ tests. It was hypothesized that (a) HDA_5m_, HDA_10m_, and HDA_20m_ tests would all exhibit good test-retest reliability and could detect moderate changes in deceleration-related kinetic and kinematic metrics, and (b) deceleration-related kinetic and kinematic metrics measured from HDA_5m_, HDA_10m_, and HDA_20m_ tests would have a low level of the correlation coefficient.

## Methods

### Participants

Eighteen young male collegiate athletes (age = 23.5 ± 2.9 years, body height = 176.8 ± 5.6 cm, body mass = 75.7 ± 7.1 kg) participated in this study. All participants were in good health and had not experienced any neuromuscular or musculoskeletal injuries in the past six months. All participants received a clear explanation of the study, including the risks and benefits of participation, and written informed consent was obtained prior to testing. Ethical consent was provided by the Shanghai University of Sport Research Ethics Committee (approval code: 102772023RT102; approval date: 24 October 2023) and in accordance with the Helsinki Declaration.

### Design and Procedures

**Figure 1:**
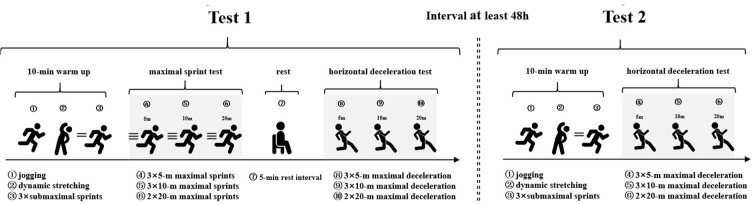
Schematic of the study design.

A test-retest study design was used to determine the reliability and sensitivity of deceleration-related metrics measured from HDA_5m_, HDA_10m_ and HDA_20m_ tests. Participants were required to complete one familiarization session and two formal test sessions for data collection (Figure 1).

A familiarization session was conducted 48 h prior to the first test session, to help participants familiarize with the test requirements. The two test sessions were separated by 48 h and set at the same time of day (9 a.m. to 5 p.m.), in order to control biological rhythm and minimize the effects of residual fatigue. In the first test session, participants' anthropometric metrics were collected, followed by the completion of 5-m, 10-m, and 20-m maximal sprint tests, and also the HDA tests with the same distances. In the second test session, participants were only required to perform HDA_5m_, HDA_10m_ and HDA_20m_ tests. Test-retest reliability and sensitivity were determined by comparing the measures obtained in each of the formal test sessions.

### Measures

#### Maximal Horizontal Sprint Test

All participants were instructed to perform three maximal effort 5-m and 10-m sprints, as well as two maximal effort 20-m sprints, with one-minute rest between trials. Participants had to wear the same shoes (Run Cushion Grip, Decathlon, France) to complete all tests. A total of eight trials were assessed using the *MySprint* app, which has been established as a reliable and valid tool for recording sprint time (Romero-Franco et al., 2017). Participants began with a 10-min warm-up including jogging, dynamic stretching and three sub-maximal 20-m sprints. Following this, formal tests were administered in specific order, including three 5-m sprint tests, three 10-m sprint tests, and two 20-m sprint tests. Participants began in a staggered-stance position with their right thumb on the ground. When ready, participants started on their own and sprinted as fast as possible until they exceeded the finish line. Finish lines of the 5-m, 10-m and 20-m sprints were clearly marked on the ground. To capture the entire sprint test, an iPhone 12 (Apple Inc., USA) was set up on a tripod at the finish line. The beginning and the end of the test were identified as the first frame in which their right thumb left the ground and the first frame in which their pelvis aligned with the marker at the finish line, respectively. The best 5-m, 10-m and 20-m sprint times in seconds (s) were recorded as the criterion time for the following HDA test.

#### Horizontal Deceleration Ability Test

The HDA test in this study was adapted from the acceleration-deceleration ability test developed by Harper et al. (2023), by including a 5-m and a 10-m distance in addition to the original 20-m linear sprints, while keeping the rest of the test procedure the same. Participants were instructed to start in a staggered stance and sprint as fast as possible over the mark at 5 m, 10 m and 20 m, before performing a maximal horizontal deceleration. Then, participants were asked to backpedal to the finish line, to create a clear “stop” and signify the end of deceleration. To ensure that participants decelerated as close to the specified distance as possible, any 5-m, 10-m or 20-m sprint time during the HDA tests that was 5% greater than the individual’s best time was excluded from the analysis. The radar device (Stalker ATS II, Applied Concepts, Inc., Dallas, TX, USA) was used to record the instantaneous horizontal velocity (m/s), distance (m), and time (s) throughout all phases of the test, with a sampling frequency of 47 Hz. The radar gun was mounted on a tripod, 1 m above the ground, and positioned 5 m behind the start line. All raw data were processed in Stalker ATS software (Version 5.0, Applied Concepts, Inc., Dallas, TX, USA) following the procedures outlined by Harper et al. (2023) and then exported as excel file for further analysis.

**Figure 2:**
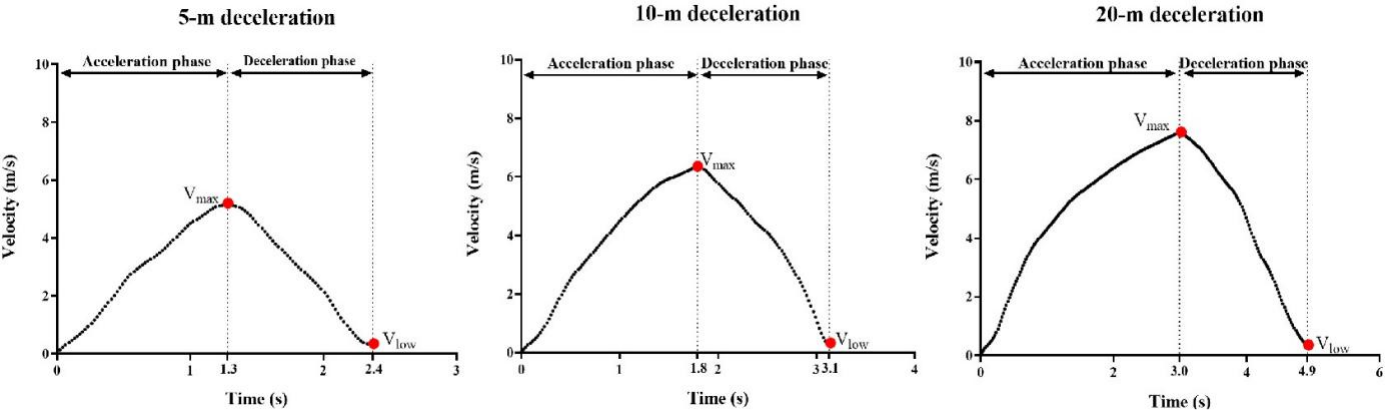
The velocity-time profile of horizontal deceleration for HDA_5m_, HDA_10m_ and HDA_20m_ tests. V_max_ = maximum velocity defining the start of the deceleration phase; V_low_ = lowest velocity defining the end of the deceleration phase

The time point of the maximum velocity (V_max_) during the HDA_5m_, HDA_10m_ and HDA_20m_ was regarded as the beginning of the deceleration phase, and the time point of the lowest velocity following V_max_ was regarded as the end of the deceleration phase (Figure 2). The formulas and description of deceleration-related metrics used in this study were in line with the previous study by Harper (2023), and are shown in Table 1. Participants were asked to perform three trials for HDA_5m_ and HDA_10m_, and two trials for HDA_20m_ tests at each test session, with the highest DEC_Ave_ of each trial used for analysis of test-retest reliability. In addition, the average values of each metrics measured from sessions 1 and 2 was used for correlation analysis.

**Table 1:** The formulas and description of deceleration-related kinematic and kinetic metrics.

	Variables	Units	Formulation	Description
Kinematic	Maximum approach velocity (V_max_)	m/s	-	The beginning of the deceleration phase.
	Distance-to-stop (DTS)	m	D _final_ – D _initial_	Distance covered from the start to the end of the deceleration phase.
	Time-to-stop (TTS)	s	T _final_ – T _initial_	Time taken from the start to the end of the deceleration phase.
	Deceleration (DEC_Ave_)	m/s^2^	(V _final_ – V _initial_) / (T _final_ – T _initial_)	The average rate of change of velocity during the deceleration phase.
Kinetic	Horizontal braking force (HBF)	N	(m × a) + Fair F air = (0.2025 × height^0.725^ × mass^0.425^) × 0.266	The braking force generated from the start to the end of the deceleration phase, calculated by the average of all instantaneous HBF values.
	Horizontal braking power (HBP)	W	HBF × v	The braking power generated from the start to the end of the deceleration phase, calculated by the average of instantaneous HBP multiply by velocity.
	Horizontal braking impulse (HBI)	N•s	(V _final_ – V _initial_) × body mass	The total of horizontal braking impulse generated from the start to the end of the deceleration phase.

Note: The above formulation and description were all in accordance with previous research (Harper et al., 2023).

### Statistical Analysis

Descriptive statistics were calculated as mean ± standard deviations (SD). The Shapiro-Wilk test was used to explore the normality of data. The intraclass correlation coefficient (ICC, two-way random with absolute agreement) with 95% confidence intervals (CI) was used to reflect the agreement between sessions 1 and 2. The ICC was interpreted as follows: > 0.9 = excellent, 0.75–0.9 = good, 0.5–0.74 = moderate, < 0.50 = poor (Koo and Li, 2016). The coefficient of variation (CV) was used to reflect the inter-subject variation in the repeated measures, and was interpreted as: > 15% poor, 10–15% moderate, 5–10% good, and < 5% excellent (Harper et al., 2023). Based on a previous study by Harper et al. (2023), overall reliability was interpreted using both the ICC and the CV scales as follows: ICC > 0.9 and CV% < 5 = excellent, ICC = 0.75 to 0.9 and CV% < 10 = good, ICC < 0.75 or CV% > 10 = moderate, ICC < 0.75 and CV% < 10 = poor.

The sensitivity of deceleration-related metrics was assessed by the standard error of measurement (SEM) and the smallest worthwhile change (SWC). The following formula: SD _pooled_ × √(1 − ICC) was used to calculate the SEM, where the SD _pooled_ represented the pooled SD for the variables of trial 1 and trial 2 (Weir, 2005). The SWC was calculated based on the between-subject SD by either 0.2 (SWC_0.2_) or 0.5 (SWC_0.5_), which represented the small and moderate effect, respectively (Lockie et al., 2013). When SEM < SWC, the ability of the test to detect a change was “good”, whereas when SEM = SWC, then the test was “satisfactory”, and when SEM > SWC, then the test was rated as “marginal” (Hopkins, 2000). Furthermore, to investigate the correlations among horizontal deceleration-related variables obtained from HDA_5m_, HDA_10m_ and HDA_20m_ tests, Pearson’s correlation coefficients (*r*) were calculated. The strength of *r* was interpreted as: 0.11–0.29 = small, 0.30–0.49 = moderate, 0.50–0.69 = large, 0.70–0.89 = very large, and ≥0.90 = almost perfect (Harper et al., 2022b). In order to avoid the potential type 1 errors when performing multiple correlations, the Bonferroni-adjusted *p*-values and their significance levels were used. All statistical analyses were performed using STATA (StataCorp 2019, Stata Statistical Software: Release 16. College Station, TX, USA: StataCorp LLC).

## Results

The deceleration-related kinematic and kinetic metrics collected from HDA_5m_, HDA_10m_ and HDA_20m_ tests are depicted in Tables 2 and 3. All correlation analyses are shown in Table 4.

**Table 2:** Test-retest reliability and sensitivity of deceleration-related metrics collected from HDA_5m_, HDA_10m_ and HDA_20m_ tests.

		Metrics	Test 1	Test 2	ICC	CV	rating
Kinematic	5-m	V_max_ (m/s)	5.31 ± 0.30	5.33 ± 0.31	0.84 (0.62–0.94)	1.90	good
		DTS (m)	3.03 ± 0.41	3.02 ± 0.44	0.75 (0.42–0.89)	5.81	good
		TTS (s)	1.01 ± 0.09	1.02 ± 0.11	0.76 (0.47–0.90)	3.27	good
		DEC_Ave_ (m/s^2^)	−4.74 ± 0.42	–4.76 ± 0.44	0.95 (0.87–0.98)	1.66	excellent
	10-m	V_max_ (m/s)	6.61 ± 0.29	6.59 ± 0.35	0.90 (0.76–0.96)	1.09	excellent
		DTS (m)	5.04 ± 0.55	4.97 ± 0.67	0.78 (0.51–0.91)	4.54	good
		TTS (s)	1.35 ± 0.13	1.34 ± 0.14	0.80 (0.54–0.92)	3.67	good
		DEC_Ave_ (m/s^2^)	–4.55 ± 0.36	–4.58 ± 0.37	0.92 (0.80–0.97)	1.55	excellent
	20-m	V_max_ (m/s)	7.70 ± 0.33	7.67 ± 0.29	0.81 (0.56–0.92)	1.45	good
		DTS (m)	7.76 ± 0.83	7.92 ± 0.91	0.44 (–0.03–0.74)	6.37	moderate
		TTS (s)	1.67 ± 0.15	1.72 ± 0.16	0.57 (0.18–0.81)	5.15	moderate
		DEC_Ave_ (m/s^2^)	–4.21 ± 0.47	–4.12 ± 0.42	0.79 (0.53–0.92)	3.69	good
Kinetic	5-m	HBF (N)	–357 ± 35	–358 ± 34	0.95 (0.87–0.98)	1.67	excellent
		HBP (W)	–1125 ± 166	–1145 ± 155	0.89 (0.73–0.96)	3.69	good
		HBI (N•s)	359 ± 34	367 ± 42	0.84 (0.61–0.94)	2.92	excellent
	10-m	HBF (N)	–343 ± 37	–345 ± 40	0.96 (0.89–0.98)	1.55	excellent
		HBP (W)	–1323 ± 180	–1320 ± 191	0.93 (0.83–0.97)	3.01	excellent
		HBI (N·s)	464 ± 50	463 ± 40	0.86 (0.66–0.94)	2.78	excellent
	20-m	HBF (N)	–323 ± 41	–316 ± 34	0.82 (0.59–0.93)	3.69	good
		HBP (W)	–1454 ± 253	–1402 ± 240	0.86 (0.65–0.94)	5.08	good
		HBI (N·s)	531 ± 58	534 ± 48	0.79 (0.51–0.91)	3.62	good

Note: V_max_ = maximum approach velocity; DTS = distance to stop; TTS = time to stop; DEC_Ave_ = average deceleration; HBF = horizontal braking force; HBP = horizontal braking power; HBI = horizontal braking impulse

**Table 3:** The sensitivity of deceleration-related kinematic and kinetic metrics measured from HDA_5m_, HDA_10m_ and HDA_20m_ tests.

		Metrics	SEM	SWC_0.2_	rating	SWC_0.5_	rating
Kinematic	5-m	V_max_ (m/s)	0.12	0.06	marginal	0.15	good
		DTS (m)	0.21	0.08	marginal	0.21	good
		TTS (s)	0.05	0.02	marginal	0.05	satisfactory
		DEC_Ave_ (m/s^2^)	0.10	0.09	marginal	0.21	good
	10-m	V_max_ (m/s)	0.10	0.06	marginal	0.16	good
		DTS (m)	0.28	0.12	marginal	0.30	good
		TTS (s)	0.06	0.03	marginal	0.07	good
		DEC_Ave_ (m/s^2^)	0.10	0.07	marginal	0.18	good
	20-m	V_max_ (m/s)	0.13	0.06	marginal	0.15	good
		DTS (m)	0.64	0.17	marginal	0.43	marginal
		TTS (s)	0.10	0.03	marginal	0.06	marginal
		DEC_Ave_ (m/s^2^)	0.20	0.09	marginal	0.22	good
Kinetic	5-m	HBF (N)	7.65	6.84	marginal	17.10	good
		HBP (W)	52.80	31.84	marginal	79.59	good
		HBI (N•s)	15.26	7.56	marginal	18.90	good
	10-m	HBF (N)	7.58	7.58	satisfactory	18.94	good
		HBP (W)	48.47	36.64	marginal	91.60	good
		HBI (N•s)	16.98	8.95	marginal	22.38	good
	20-m	HBF (N)	15.88	7.48	marginal	18.71	good
		HBP (W)	91.47	48.89	marginal	122.23	good
		HBI (N·s)	24.41	10.53	marginal	26.32	good

Note: V_max_ = maximum approach velocity; DTS = distance to stop; TTS = time to stop; DEC_Ave_ = average deceleration; HBF = horizontal braking force; HBP = horizontal braking power; HBI = horizontal braking impulse

**Table 4:** The relationships between kinematic and kinetic metrics from HDA_5m_, HDA_10m_ and HDA_20m_ tests.

	Comparisons	*r*	95% CI	rating	*p*_adjust_
**Kinematic**					
DTS (m)	HDA_5m_ *vs*. HDA_10m_	0.19	(–0.31–0.60)	small	1.000
	HDA_5m_ *vs*. HDA_20m_	0.18	(–0.32–0.59)	small	1.000
	HDA_10m_ *vs*. HDA_20m_	0.65	(0.26–0.86)	large	0.010*
TTS (s)	HDA_5m_ *vs*. HDA_10m_	0.34	(0.03–0.70)	moderate	0.488
	HDA_5m_ *vs*. HDA_20m_	0.39	(0.10–0.63)	moderate	0.337
	HDA_10m_ *vs*. HDA_20m_	0.76	(0.57–0.94)	very large	**0.001**
DEC_Ave_ (m/s^2^)	HDA_5m_ *vs*. HDA_10m_	0.44	(0.01–0.81)	moderate	0.200
	HDA_5m_ *vs*. HDA_20m_	0.61	(0.40–0.82)	large	0.022*
	HDA_10m_ *vs*. HDA_20m_	0.71	(0.43–0.91)	very large	0.003*
**Kinetic**					
HBF (N)	HDA_5m_ *vs*. HDA_10m_	0.59	(0.12–0.88)	large	0.029*
	HDA_5m_ *vs*. HDA_20m_	0.64	(0.27–0.85)	large	0.013*
	HDA_10m_ *vs*. HDA_20m_	0.76	(0.53–0.91)	very large	0.001*
HBP (W)	HDA_5m_ *vs*. HDA_10m_	0.81	(0.34–0.96)	very large	0.000*
	HDA_5m_ *vs*. HDA_20m_	0.77	(0.20–0.91)	very large	0.001*
	HDA_10m_ *vs*. HDA_20m_	0.87	(0.58–0.96)	very large	0.000*
HBI (N·s)	HDA_5m_ *vs*. HDA_10m_	0.89	(0.79–0.96)	very large	0.000*
	HDA_5m_ *vs*. HDA_20m_	0.82	(0.63–0.92)	very large	0.000*
	HDA_10m_ *vs*. HDA_20m_	0.88	(0.74–0.95)	very large	0.000*

Note: DTS = distance to stop; TTS = time to stop; DEC_Ave_ = average deceleration; HBF = horizontal braking force; HBP = horizontal braking power; HBI = horizontal braking impulse; * and bold font = p < 0.05

### Reliability and Sensitivity

All kinematic variables from HDA_5m_ and HDA_10m_ tests showed moderate to excellent reliability (HDA_5m_: CV = 1.66%–5.81%, ICC = 0.74–0.95; HDA_10m_: CV = 1.09%–4.54%, ICC = 0.78–0.92) and were able to detect SWC_0.5_. For HDA_20m_, V_max_ (CV = 1.45%, ICC = 0.81) and DEC_Ave_ (CV = 3.69%, ICC = 0.79) had good reliability, while DTS (CV = 6.37%, ICC = 0.44) and TTS (CV = 5.15%, ICC = 0.57) showed moderate reliability, and only V_max_ and DEC_Ave_ could detect SWC_0.5_. All kinetic metrics of HDA_5m_ and HDA_10m_ showed good to excellent inter-test reliability (HDA_5m_: CV = 1.67%–3.69%, ICC = 0.89–0.95; HDA_10m_: CV = 1.55%–3.01%, ICC = 0.93–0.96), with sufficient sensitivity to detect SWC_0.5_. For HDA_20m_, the overall reliability was moderate to good (CV = 3.69%–5.54%, ICC = 0.59–0.86), and all metrics were able to detect the SWC_0.5_.

### Correlations Analyses

Significant correlations were found in four out of ninecomparisons for kinematic metrics (Table 4). For HDA_5m_, only DEC_Ave_ had a significant large correlation with HDA_20m_ (*r* = 0.61, *p* < 0.05). Significant and large-to-very large correlations were also found between HDA_10m_ and HDA_20m_ in DTS (*r* = 0.65, *p* < 0.05), TTS (*r* = 0.76, *p* < 0.05) and DEC_Ave_ (*r* = 0.71, *p* < 0.05). All comparisons of the kinetic metrics showed significant large-to-very large correlations (*r* = 0.59 to 0.89, *p* < 0.05).

## Discussion

To our best knowledge, this is the first study to examine the test-retest reliability, sensitivity, and correlations between deceleration kinematic and kinetic metrics measured from HDA_5m_, HDA_10m_ and HDA_20m_ tests. The major findings are that: (1) all kinematic and kinetic metrics from HDA_5m_, HDA_10m_ and HDA_20m_ tests exhibited good to excellent test-retest reliability and could detect moderate changes in deceleration performance, except the DTS and TTS of the HDA_20m_ test, (2) deceleration-related kinematic metrics of the HDA_5m_ test had non-significant and small-to-moderate correlations with HDA_10m_ and HDA_20m_ tests, except the DEC_Ave_ between HDA_5m_ and HDA_20m_, (3) all comparisons of kinetic metrics among three different HDA tests had significant and large-to-very large correlations.

The importance of horizontal deceleration in multi-directional sport competitions has been increasingly recognized due to its significance for athletic performance and injury-risk reduction (Harper et al., 2019; Harper and Kiely, 2018). However, so far only two studies have explored the reliability and sensitivity of deceleration-related metrics when deceleration was performed from longer sprinting distances (i.e., 15 m and 20 m) (Ashton and Jones, 2019; Harper et al., 2023). Similarly to the work conducted by Harper et al. (2023), we investigated the test-retest reliability and sensitivity of deceleration-related metrics measured from a 20-m sprint and identified that DEC_Ave_, HBF, HBP and HBI had moderate to good test-retest reliability and were sensitive at detecting moderate changes in deceleration performance. These findings are in agreement with Harper et al. (2023) who also reported good inter-day reliability for HBF and HBP. As such, sports science, coaching and medical practitioners can use these metrics to evaluate their athletes’ deceleration performance from an applied performance environment with higher sprinting speeds and distances.

Further to the data from the HDA_20m_ test, this study also explored the test-retest reliability and sensitivity of deceleration kinematic and kinetic metrics when deceleration was performed from shorter sprinting distances (i.e., 5 m and 10 m). Philipp et al. (2023a, 2023b) have previously reported CV values of 3.01–11.46% for horizontal deceleration metrics (i.e., DEC_Avg_, TTS, HBI) obtained from HDA tests performed with 10 and 20-yard sprints in female softball players (Philipp et al., 2023a), in addition to the HDA_10m_ test (CVs: 3.01–16.66%) performed by female handball players (Philipp et al., 2023b). Nevertheless, it should be pointed out that those studies have some limitations, and the data may not provide sufficient information regarding the reliability of deceleration metrics. For instance, the first study only reported CV values for the test as a whole, thus the intra-test reliability of each metric remained uncertain (Philipp, et al., 2023a). In the second study, the sample size (*n* = 11) and the repetitions of each test (*n* = 2) were arguably insufficient, combined with the novelty of the task, resulting in higher CVs during the HDA_10m_ test, which reached 16.66% (Philipp et al., 2023b). Therefore, this is the first study to investigate and establish the test-retest reliability and sensitivity of deceleration kinetic and kinematic metrics performed with shorter sprinting distances, with a requirement to be within 5% of their best linear sprint time before commencing a maximal deceleration at the 5-m and 10-m points. A novel and important finding was that all deceleration kinetic and kinematic metrics had good-to-excellent reliability and were sensitive to detecting moderate changes (SEM < SWC_0.5_) in deceleration performance (Table 3). This could be attributed to the fact that athletes had less momentum (V × body mass) in the forward direction to contend with when decelerating from the HDA_5m_ and HDA_10m_ tests. Thus, it made athletes’ horizontal deceleration performance more “controllable” resulting in high repeatability of these metrics when measured between session 1 and session 2. Consequently, practitioners and researchers can utilize those metrics to evaluate an athlete’s HDA from specific training.

Another key finding of this study is that correlations among the horizontal deceleration-related metrics at HDA_5m_, HDA_10m_ and HDA_20m_ tests differed (Table 4). Specifically, metrics measured from the HDA_5m_ test exhibited unique kinematic profiles, with the DTS and TTS having non-significant and small to moderate correlations with those measured in HDA_10m_ and HDA_20m_ tests. This is potentially important because it suggests that findings from previous studies may not be applicable to deceleration abilities performed with lower sprinting distances and speeds (i.e., HDA_5m_ test). For example, lower extremity muscle qualities such as knee flexor and knee extensor concentric strength (Harper et al., 2021) and the ability to quickly produce high knee extensor eccentric torque (Zhang et al., 2021) have been reported to be associated with deceleration abilities when performed with a 20-m sprinting distance. Therefore, further exploration of the determinants of short-distance deceleration performance is necessary, especially for athletes who perform frequently a large number of short-distance deceleration tasks.

On the other hand, all kinematic and kinetic metrics of the HDA_10m_ test showed significant and large-to-very large correlations with the HDA_20m_ test, which indicates that an athlete with good horizontal deceleration performance at the 10-m distance may also have good deceleration performance during the 20-m HDA test. It is in line with previous research where significant associations of DEC_Avg_ and HBI were identified between HDA_10yard_ and HDA_20yard_ tests (Philipp et al., 2023a). However, the correlation of DEC_Avg_ between the HDA_10yard_ test and the HDA_20yard_ test was found to be negative (*r* = −0.443), which contrasts with this study. By comparing the data of the present study with those of Philipp et al. (2023a), it is clear that the DEC_Avg_ values of the HDA_10m_ test are noticeably larger than of the HDA_10yard_ test (HDA_10m_ vs. HDA_10yard_: −4.56 ± 0.36 m/s^2^ vs. −3.26 ± 0.30 m/s^2^), which reveals that participants in this study generated greater velocity reductions during the deceleration phase. This discrepancy may be attributed to the fact that participants in our study (male amateur university athletes) differed from those in the previous study, who were NCAA Division I female softball players. This may suggest that gender, the level of competition, and engagement in different sports may impact horizontal deceleration performance.

Whilst the current study provides novel information on the assessment of horizontal deceleration, a potential limitation is that the participants recruited were all male amateur university athletes, which means that the current conclusions may not be applicable to elite athlete or female populations. Additionally, participants in the current study competed in a range of multi-directional sports, such as basketball, soccer, and badminton, thus caution should be taken when adapting the results of this study to athletes in a specific sport, because proficiency in motor performance may differ between different sporting populations. Finally, this study can only verify that the kinematic and kinetic metrics measured from the HDA_5m_ and HDA_10m_ tests are both reliable and sensitive. This means that the conclusions of this study only apply to short-distance horizontal deceleration tests. Future research can further explore whether this method is also suitable for quantifying deceleration ability in change-of-direction tests.

In conclusion, our findings indicate that both HDA_5m_ and HDA_10m_ tests present good to excellent test-retest reliability and are sensitive enough to detect moderate changes in horizontal deceleration performance. In addition, the unique kinematic profile of the HDA_5m_ test reveals the need for future studies to determine the physiological and biomechanical factors that could influence maximal deceleration performance obtained within shorter sprinting distances. This will provide a deeper understanding of horizontal deceleration ability and establish a theoretical basis for the practical field.

## Conclusions

The findings of the present study indicate that short-distance HDA tests demonstrate good test-retest reliability and sufficient sensitivity to detect the moderate changes in deceleration performance. Researchers and practitioners can therefore analyze both kinematic and kinetic metrics obtained from HDA_5m_ and HDA_10m_ tests to assess and monitor training induced changes in an athlete’s deceleration ability. In addition, considering the unique kinematic characteristics observed in the HDA_5m_ test, it is crucial to emphasize the importance of developing specific training programs aimed at helping athletes decelerate in the shortest time and distance possible from lower horizontal velocities.
